# The Expression of Chemokines Is Downregulated in a Pre-Clinical Model of TTR V30M Amyloidosis

**DOI:** 10.3389/fimmu.2021.650269

**Published:** 2021-05-19

**Authors:** João Moreira, Susete Costelha, Margarida Saraiva, Maria João Saraiva

**Affiliations:** ^1^ i3S – Instituto de Investigação e Inovação em Saúde, University of Porto, Porto, Portugal; ^2^ IBMC – Instituto de Biologia Molecular e Celular, University of Porto, Porto, Portugal; ^3^ ICBAS - Instituto de Ciências Biomédicas Abel Salazar, University of Porto, Porto, Portugal

**Keywords:** transthyretin, chemokines, Schwann cell, immune regulation, familial amyloidotic polyneuropathy

## Abstract

Inflammation is a hallmark of several neurodegenerative disorders including hereditary amyloidogenic transthyretin amyloidosis (ATTRv). ATTRv is an autosomal dominant neurodegenerative disorder with extracellular deposition of mutant transthyretin (TTR) aggregates and fibrils, particularly in nerves and ganglia of the peripheral nervous system. Nerve biopsies from ATTRv patients show increased cytokine production, but interestingly no immune inflammatory cellular infiltrate is observed around TTR aggregates. Here we show that as compared to Wild Type (WT) animals, the expression of several chemokines is highly downregulated in the peripheral nervous system of a mouse model of the disease. Interestingly, we found that stimulation of mouse Schwann cells (SCs) with WT TTR results in the secretion of several chemokines, a process that is mediated by toll-like receptor 4 (TLR4). In contrast, the secretion of all tested chemokines is compromised upon stimulation of SCs with mutant TTR (V30M), suggesting that V30M TTR fails to activate TLR4 signaling. Altogether, our data shed light into a previously unappreciated mechanism linking TTR activation of SCs and possibly underlying the lack of inflammatory response observed in the peripheral nervous system of ATTRv patients.

## Introduction

Amyloid disorders are a heterogeneous group of diseases that can either be focal or systemic. They are due to the extracellular deposition of insoluble misfolded proteins, ultimately disrupting normal tissue function (1). Among them, transthyretin (TTR) amyloidosis is the most common form of hereditary autosomic dominant systemic amyloidosis, in which TTR point mutations result in deposition of amyloidogenic species in different tissues (2). Recently this disease has been designated as ATTRv (3). Over 150 mutations in the primary sequence of TTR have been identified, most of which are associated with peripheral neuropathy as the main clinical manifestation. Often, cardiomyopathy and carpal tunnel syndrome occur as well (4, 5). The most common TTR mutation in ATTRv results from an exchange of a methionine for a valine at position 30 (TTR V30M) (6). The expression of TTR was recently reported in Schwann cells (SCs) of the sciatic nerve (7), but this protein is mainly synthesized in the liver and choroid plexus of the brain being secreted into the blood stream and cerebrospinal fluid, respectively (8, 9).

V30M related ATTRv was originally described as FAP (Familial Amyloidotic Polyneuropathy) by Corino de Andrade in northern Portugal and, despite being rare, the disease is distributed worldwide (10, 11). It is a fatal neurodegenerative disorder, characterized by the extracellular deposition of aggregates and fibrils of mutant forms of TTR, particularly in the nerves and ganglia of the peripheral nervous system (PNS) (2). Pro-inflammatory mechanisms are upregulated in ATTRv, especially in endoneurial axons, with increased expression of TNF-α and IL-1β since earlier stages of disease, increasing with the ongoing neurodegenerative process (12). Therefore, infiltration of inflammatory cells in response to TTR deposition in tissues of ATTRv patients would be expected. Surprisingly, despite local increased cytokine production, such as IL-1β and TNF-α, by axons, as well as local activation of NF-kB, no immune inflammatory cellular infiltrate is observed around TTR aggregates in nerves from V30M patients, which might contribute to disease progression and suggests that mechanisms must operate to prevent or inhibit the correct innate immune response (12, 13). Furthermore, immunochemical studies of patient’s nerves suggest an impairment in the ability to express chemokines and neurotrophic factors important to drive tissue regeneration (12, 14). Additionally, after nerve injury in animal models carrying the V30M mutation (15, 16) a downregulated innate immune response was observed when compared to WT mice. More specifically, a decreased expression of several cytokines and chemokines important for the recruitment of immune cells like macrophages was found (17).

All these observations correlated with the downregulated expression of chemokines, including CCL20 found in previous microarrays (18) in the peripheral nerve of a TTR V30M transgenic mouse model, prompted us to investigate the chemokine response activation in ATTRv pathogenesis.

## Material and Methods

### Animals

All animal experiments were carried out in accordance with National and European Union guidelines for the care and handling of laboratory animals, and were performed in compliance with the institutional guidelines and recommendations of the Federation for Laboratory Animal Science Association (FELASA) and approved by the National Authority for Animal Health (DGAV; Lisbon, Portugal). Transgenic mice for human TTR V30M, in the Sv/129 and endogenous TTR-null background, with heterozygous deletion of the gene encoding transcription factor Hsf-1 (labeled as HSF/V30M) (16) were analyzed at 6 and 20 months of age (n=6 of each age). WT animals heterozygous for Hsf-1 deletion, in the Sv/129 background, were used as controls (n=6 of each age).

Animals were housed in a controlled temperature room, maintained under a 12-h light/dark period, with water and food ad libitum and then sacrificed with a lethal injection of a premixed solution containing ketamine (75 mg/kg) plus medetomidine (1 mg/kg). Plasma and sciatic nerve were collected. They were frozen at −80°C, or fixed in formalin.

### Recombinant Transthyretin

Recombinant WT TTR and TTR variants, namely V30M, I68L, T119M and I84S TTR were produced in a bacterial expression system and purified as previously described (19).

### Recombinant Transthyretin Purification

Prior to use, endotoxins were removed from WT TTR and V30M, I68L, T119M and I84S TTR mutations. Briefly, Detoxi-Gel endotoxin resin (Thermo Scientific, Waltham, MA, catalog#20339) was packed appropriately in a chromatography column and was allowed to settle for 30 minutes. Detoxi-Gel Resin was regenerated by washing with five resin-bed volumes of 1% sodium deoxycholate (Sigma-Aldrich, Germany, catalog#D6750-10G), followed by 3-5 resin-bed volumes of pyrogen-free buffer to equilibrate the resin. Then, the samples were applied to the column and for greater efficiency the samples were incubated with the resin for 1 hour at room temperature. Pyrogen-free buffer was used to collect the flow-through. The protein quantification after elution was assessed by Bradford method (20).

### Dot Blot for Detecting Protein Aggregation

Aliquots corresponding to equal amounts of WT TTR and V30M, I68L, T119M and I84S TTR mutations (500 ng) were blotted onto a 0.2-μm pore cellulose acetate membrane and it was left to dry at room temperature for few hours. TTR was immunodetected using an antibody targeting TTR aggregates produced in our lab, mouse CE11 antibody (1:20) followed by anti-mouse horseradish peroxidase antibody (1:5000) and enhanced chemiluminescence visualization (BioRad). No aggregates were detected in all TTR samples.

### Schwann Cell Primary Culture

Mouse Schwann cell primary cultures were obtained from ScienCell Research Laboratories™ (San Diego, CA, catalog #M1700) and were cultured according to the manufacturer’s instructions. Briefly, cells were cultured in Schwann cell medium, supplemented with 5% fetal bovine serum, 1% Schwann cell growth supplement and 1% penicillin/streptomycin (all from ScienCell Research Laboratories). After monolayer propagation in T-75 flasks at 37°C in a 5% CO2 humidified chamber, 5×10^5^ cells were seeded in 12-well plates and incubated with WT or V30M TTR, both at 5 μM in triplicates. In a different experiment Schwann cells were incubated with Cli-095 (InvivoGen, San Diego, CA, catalog #tlrl-cli95) and sRage which was isolated from bacteria inclusion bodies (21), antagonists for TLR4 and Rage respectively, before a second incubation with WT or V30M TTR. Twenty-four hours after stimulation, supernatants were collected and cell lysates were prepared in trizol (Invitrogen, Waltham, MA, catalog #15596026) and assayed for the expression of proinflammatory cytokines by RT-PCR. Unstimulated cells were also used as controls.

### RNA Extraction, cDNA Conversion, and Real-Time PCR

RNA from sciatic nerves (n=6 per group) was isolated with RNeasy Mini columns, following the manufacturer′s instructions (Qiagen, Hilden, Germany, catalog #74804). RNA from mouse Schwann cells culture were isolated by phenol extraction (Trizol, Invitrogen). First-strand complementary DNA (cDNA) was synthesized using the SuperScript double-stranded cDNA Kit (Invitrogen) and quantitative real-time PCR performed using the iQ Syber Green Super Mix (Bio-Rad). Samples were run in duplicate and results analyzed by the Bio-Rad iQ5 software. Glyceraldehyde 3-phosphate dehydrogenase (Gapdh) was used as reference gene. For the quantification of mRNA expression levels, the reaction was performed in a final volume of 20 μL containing 0.5 μL of each specific primer: mouse Gapdh forward: GCCTTCCGTGTTCCTACC, mouse Gapdh reverse: AGAGTGGGAGTTGCTGTTG; mouse CCL20 forward: TACAGACGCCTCTTCCTT, mouse CCL20 reverse: TCGTGTGAAAGATGATAGCA; mouse CCL5 forward: CAATCTTGCAGTCGTGTT, mouse CCL5 reverse: AATAGTTGATGTATTCTTGAACC; mouse CXCL5 forward: CTACGGTGGAAGTCATAG, mouse CXCL5 reverse: TTCTTTATCACAGGAGCTT; mouse CCL2 forward: GATGATCCCAATGAGTAG, mouse CCL2 reverse: AAATAAAGTTGTAGGTTCTG; mouse CCL8 forward: AGAGAATCAACAATATCCA, mouse CCL8 reverse: CTACACAGAGAGACATAC; mouse CXCL3 forward: CCAACCACCAGGCTACAG, mouse CXCL3 reverse: AACTTCTTGACCATCCTTGA; mouse CXCL2 forward: CCAACCACCAGGCTACAG, mouse CXCL2 reverse: CTTCAGGGTCAAGGCAAAC; mouse IL-1β forward: AAGATGAAGGGCTGCTTCCA, mouse IL-1β reverse: AAGGTCCACGGGAAAGACAC; mouse IFN-β forward: GCACTGGGTGGAATGAGACT, mouse IFN-β reverse: AGTGGAGAGCAGTTGAGGACA; mouse IL-6 forward: CTGTCTATACCACTTCAC, mouse IL-6 reverse: GCTTATCTGTTAGGAGAG; (all from Sigma), and 18 μL of Mix plus 1 μL of the newly synthesized cDNA. Primer sequences were designed using Beacon Designer 8 (Premier Biosoft) for all genes. Analysis of real-time PCR data was made by the comparative CT method. Individual relative gene expression values were calculated using the following formula: 2 ^− (Ct gene of interest− Ct constitutive gene)^ (22).

### RNA Sequencing

RNA from sciatic nerves (n=4) was isolated from HSF WT and HSF V30M mice with RNeasy Mini columns and first-strand complementary DNA (cDNA) was synthesized using the SuperScript double-stranded cDNA Kit as abovementioned. Subsequently, the cDNA samples were delivered to Bioinf2Bio Company for RNA sequence analysis. All reads were aligned to the Ensembl genome of *Mus musculus* reference genome GRCm38. Bioinf2Bio used the reference mouse genome, based on the strain C57BL/6J, for read alignment. The differential expression between the different groups of samples were identified by the tool *Cuffdiff* to find significant changes in gene expression across conditions. All data used in the present study are available in NCBI GEO database with the access number GSE165126.

### Western Blot Analysis

Protein was isolated from Schwann cells by homogenization with lysis buffer containing 10 μL phenylmethylsulfonyl fluoride (PMSF), 10 μL sodium orthovanadate solution and 10 μL protease inhibitor cocktail solution per mL of 1x RIPA lysis buffer (Santa Cruz Biotechnology, Santa Cruz, CA, catalog# sc-24948). Protein concentration was determined using the Bradford protein assay (Bio-Rad, Hercules, CA, catalog# 5000006). Subsequently, samples were run on a 12% polyacrylamide gel electrophoresis (SDS-PAGE) and blotted on nitrocellulose WhatmanTM membrane (GE Healthcare). Primary antibodies against Phospho-p38 (Cell Signaling, Netherlands, catalog# 4631), and α-Tubulin (Sigma, Germany, catalog# T8203) were diluted in 5% bovine serum albumin in PBS supplemented with 0.1% Tween 20 (PBS-T), overnight at 4°C. In the next day, blots were washed with PBS-T and incubated with anti-rabbit or anti-mouse conjugated to horseradish peroxidase (1:5000; The Binding Site) for 45 min at room temperature. After three washes in PBS-T, bands were visualized with enhanced chemiluminescence using the LuminataTM Crescendo (Millipore). Protein bands were quantified by densitometry using Quantity One software (Bio-Rad) and α-Tubulin quantification was used to correct for total protein loading variation.

### Enzyme-Linked Immunosorbent Assay

The levels of CCL20 (R&D Systems, Minneapolis, MN, catalog# MCC200) and CXCL2 (R&D Systems, Minneapolis, MN, catalog# MM200) in the supernatants of Schwann cells incubated with WT and V30M TTR and in plasma from HSF WT and HSF V30M mice were quantitatively determined by enzyme-linked immunosorbent assay (ELISA), according to the manufacturer′s instructions.

### Statistical Analysis

Statistical comparison of data was performed using the Student t test or one-way ANOVA with Graph Pad Prism software. Quantitative data are expressed as mean ± SEM. Statistical significance was established for p* < 0.05, p** < 0.01, and p*** < 0.001.

## Results

### The Expression of Several Chemokines Is Downregulated in the Peripheral Nervous System of HSF V30M TTR Mice

**Figure 1:**
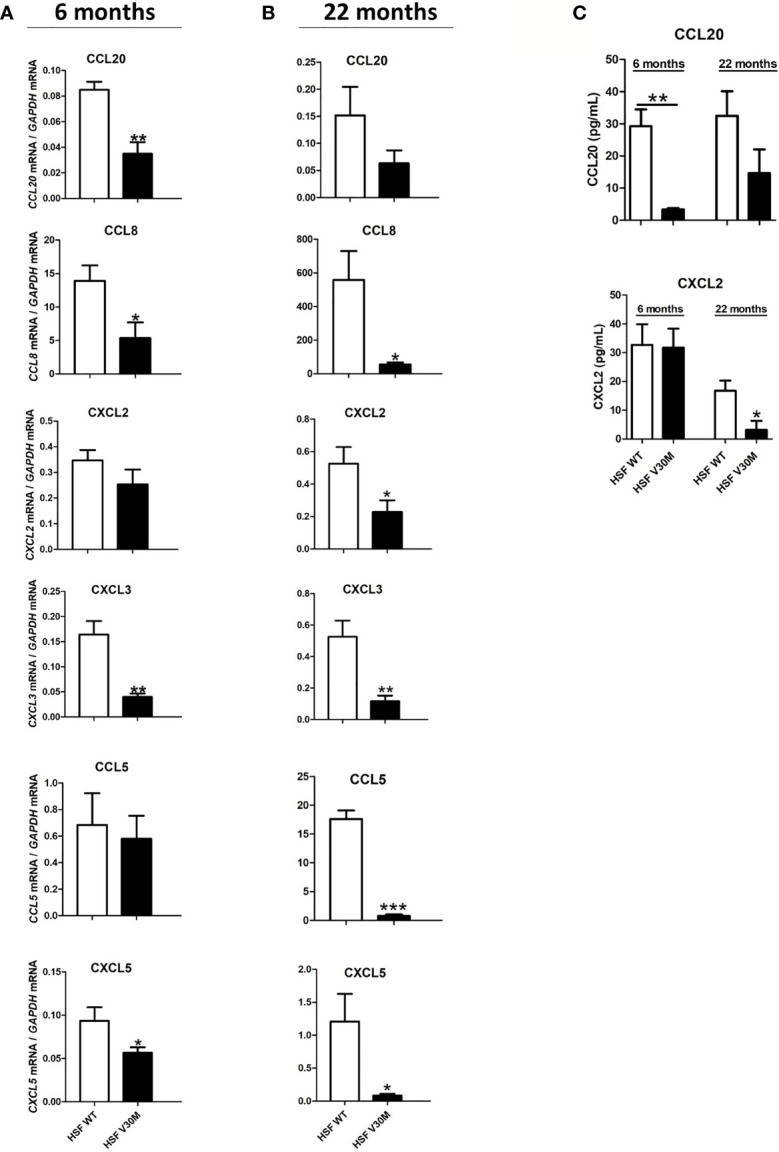
The levels of several chemokines were downregulated in an ATTRv mouse model of the disease. RNA was extracted from sciatic nerve of HSF WT and HSF V30M mice with 6 **(A)** and 20 **(B)** months of age and the levels of several chemokines were found highly downregulated in the sciatic nerve of HSF V30M mice at both ages. **(C)** Decreased plasma levels of CCL20 and CXCL2 were measured by ELISA in the serum of HSF V30M mice with 6 and 20 months of age. Data were analyzed using one-way ANOVA followed by Bonferroni post-test and represented as mean ± s.e.m (*P < 0.05; **P < 0.01; ***P < 0.001).

Despite local increased cytokine production, the recruitment of immune cells like macrophages and neutrophils, known to be important for the tissue regenerative process, is reduced in ATTRv nerve biopsies (13). We questioned if the impaired immune cell recruitment was due to a compromised chemokine response. To address this hypothesis, an RNA sequence analysis was performed in sciatic nerve from a mouse model of the disease (HSF V30M TTR mice) and the respective control group (HSF WT TTR mice) with 6 months of age. This ATTRv mouse model carries the V30M mutation in a heterozygous background for the heat shock factor 1 (Hsf-1) and deposits TTR non-fibrillar species in the peripheral nervous system (16). The expression of most chemokines was significantly downregulated in the sciatic nerve of HSF V30M TTR mice when compared to HSF WT control at 6 months of age (**Table 1**), a finding that we validated by RT-PCR (**Figure 1A**). In older animals (22 months), a similar and possibly more marked profile revealing the downregulation of chemokine expression was observed (**Figure 1B**).

**Table 1:** Chemokines found downregulated in V30M sciatic nerve compared with sciatic nerve from WT mice.

Gene Name	Symbol	Fold change
Chemokine (C-C motif) ligand 8	CCL8	2,56
Chemokine (C-C motif) ligand 5	CCL5	2,4
Chemokine (C-X-C Motif) ligand 3	CXCL3	Not detected in V30M TTR mice
Chemokine (C-C Motif) ligand 20	CCL20	Not detected in V30M TTR mice

Genes were considered up-regulated with fold-change >1.5 after class comparison, assuming significances P < 0.05.

To further validate the RNA data, we measured the levels of CCL20 and CXCL2 by ELISA in the serum of HSF WT and HSF V30M TTR mice at 6 and 20 months of age. A significant decrease in CCL20 levels from HSF V30M mice with 6 months of age comparatively with HSF WT mice was observed, but no significant differences were detected in HSF V30M mice with older age (**Figure 1C**). In contrast, a significant decrease in CXCL2 levels was observed in the serum of older, but not young, HSF V30MTTR mice as compared to HSF WT mice (**Figure 1C**). These data are in line with the RNA profiles obtained.

Collectively, our data point to a decreased expression of chemokines in a mouse model of ATTRv.

### Schwann Cells Are Activated by WT TTR, but Not by V30M TTR

SCs, the myelinating glial cells of the peripheral nervous system, play a crucial role in the generation, function and maintenance of peripheral nerves (23). In fact, SCs act as the nerve sentinel cells and have the ability to regulate the PNS immune response by secreting cytokines and chemokines, thus playing a central role in nerve repair (14, 24). Because the expression of chemokines is limited in the nerve of ATTRv mouse models and because TTR accumulates in the nerves of ATTRv, we next studied the inflammatory profile of SCs in response to WT and V30M TTR.

**Figure 2:**
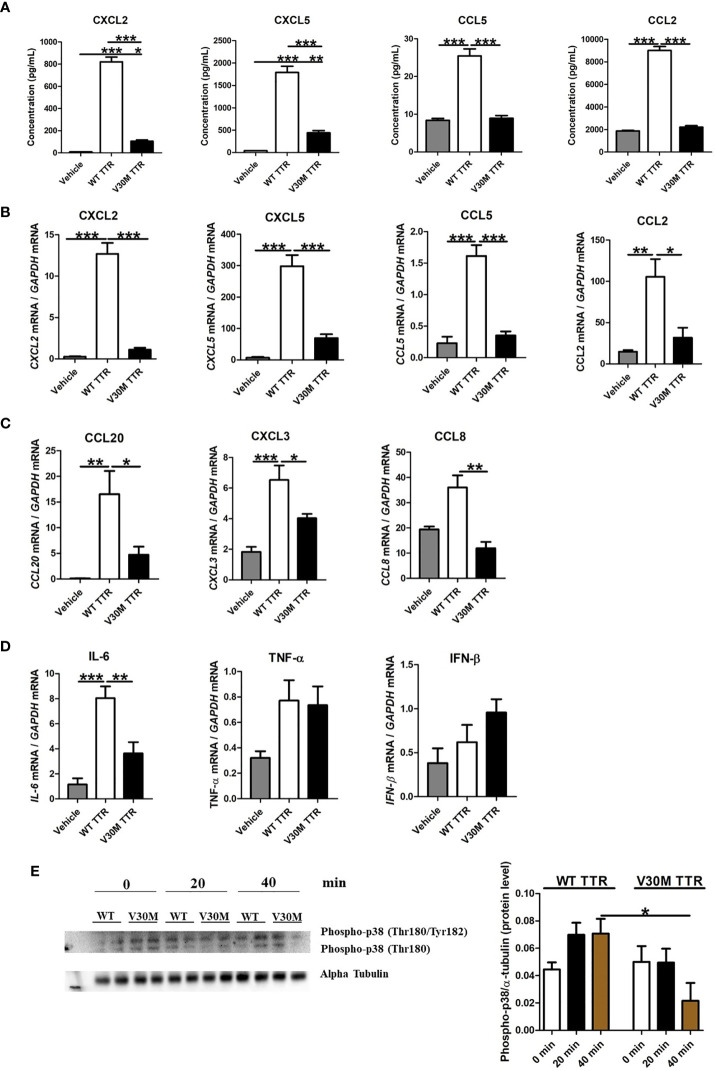
The expression of chemokines were downregulated in Schwann cells incubated with V30M TTR. **(A)** A Luminex analysis showed a decreased expression of chemokines in SCs incubated with V30M TTR when compared with SCs incubated with WT TTR for 24 hours. **(B)** RNA was extracted from SCs incubated with WT and V30M TTR and the levels of chemokines were found highly downregulated with V30M TTR incubation. **(C)** Reduced levels of CCL20, CCL8 and CXCL3 were determined by RT-PCR in SCs incubated with V30M TTR when compared to WT TTR. **(D)** The expression of cytokines were assessed by RT-PCR in SCs incubated with WT or V30M TTR. **(E)** Western blot analysis for the phosphorylation of p-38 in SCs incubated with WT or V30M TTR at different time points. Were observed two forms of Phospho-p38 corresponding to Phospho-p38 MAP Kinase dually phosphorylated at threonine 180 and tyrosine182, and with Phospho-p38 singly phosphorylated at Thr180. Data were analyzed using one-way ANOVA followed by Bonferroni post-test and represented as mean ± s.e.m (*P < 0.05; **P < 0.01; ***P < 0.001).

For that, mouse SCs were incubated with human WT or V30M TTR for 24 hours and the secretion of a series of chemokines measured by luminex. Whereas WT TTR activated SC leading to the secretion of all tested chemokines, V30M TTR failed to do so (**Figure 2A**). To understand if the observed differences resulted from a transcriptional alteration, SCs were stimulated as above, RNA was extracted, and the expression levels of the chemokines were assessed by real-time PCR. In accordance with the protein results, the expression of all tested chemokines was upregulated in SCs incubated with WT TTR, but not with V30M TTR (**Figure 2B**). Additionally, an upregulation of the transcription of CCL20, CXCL3 and CCL8, which were altered in the mouse model, was detected in SCs stimulated with WT TTR, but not with V30M TTR (**Figure 2C**). These results indicate that WT TTR, but not mutated TTR, activates SCs leading to the production of proinflammatory chemokines. Furthermore, we incubated SCs with mouse TTR as a control, measured the expression of chemokines and observed similar results between SCs incubated with mouse or human WT TTR (data not shown).

To address how broad the response of SCs to TTR is, we also measured the expression of several cytokines upon SC stimulation. The expression of IL-6 induced in SCs by WT TTR was significantly higher than that induced by V30M TTR (**Figure 2D**). Both TNF and IFN-β were poorly induced by either form of TTR (**Figure 2D**).

Lastly, we evaluated if incubation of SCs with WT or V30M TTR induced signal transduction pathways by measuring the phosphorylation of the MAP kinase p38 over time post-stimulation, by western blot analysis. MAP kinase p38 regulates multiple cellular functions, including cell proliferation, differentiation, stress response, apoptosis, and cell migration and survival, among others, by interacting with a plethora of substrates (25, 26). p38 is activated by a wide range of environmental stimuli, inflammatory cytokines, PAMPs (pathogen-associated molecular patterns), and DAMPs (danger-associated molecular patterns) (27). Whereas an increase of phosphorylated p38 was observed upon activation of SCs with WT TTR, the very opposite was observed in SCs incubated with V30M TTR (**Figure 2E**). These observations suggest that WT TTR activates SCs to produce several immune mediators, while V30M TTR does not.

### Schwann Cells Are Activated by Other Mutated TTR Variants

**Figure 3:**
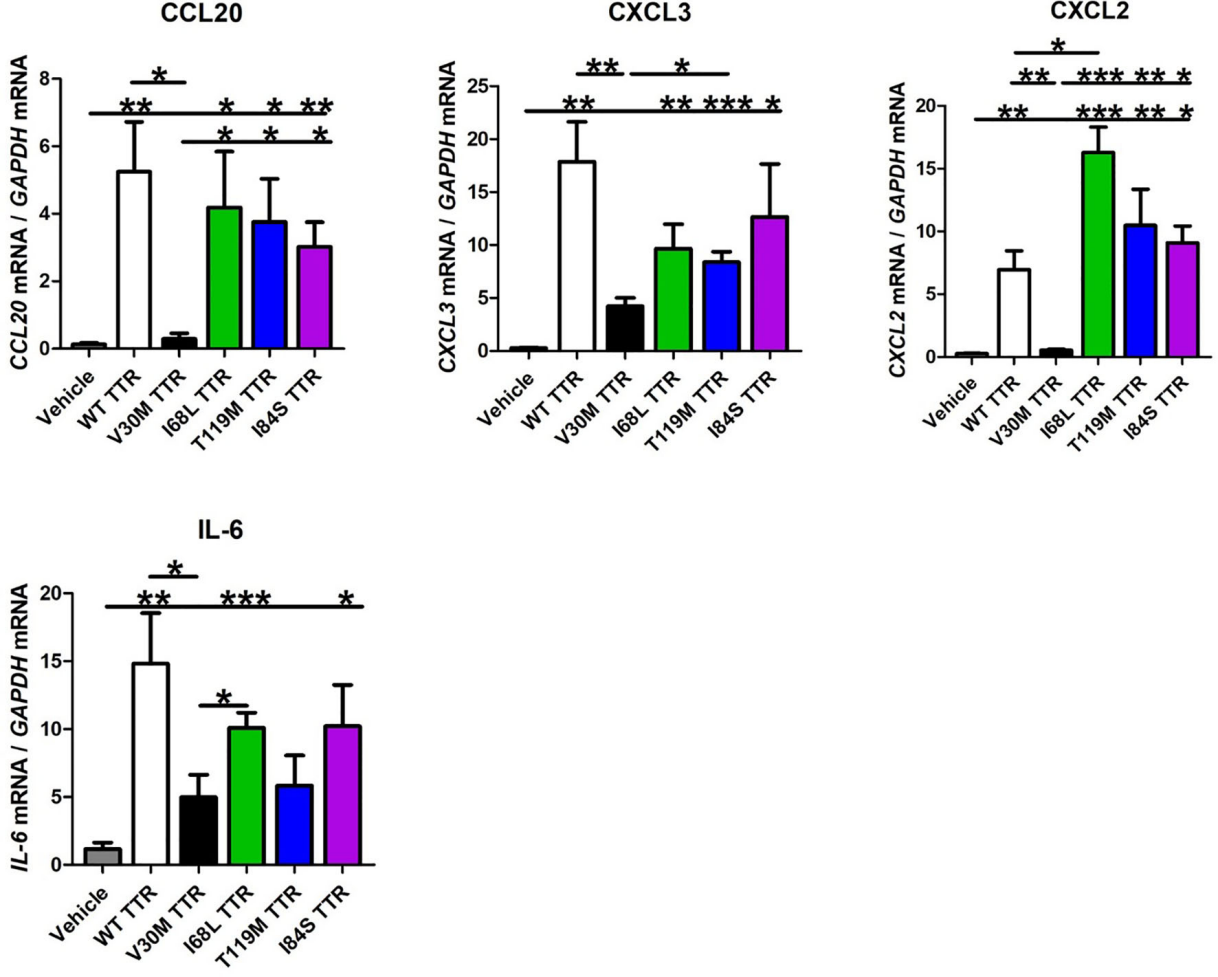
Schwann cells are activated by other TTR variants. Schwann cells were activated by different TTR pathological or non-pathological mutations, leading to an expression of chemokines globally similar to that induced by WT TTR. IL-6 expression was also similar between SCs incubated with WT or mutant TTRs. Data were analyzed using one-way ANOVA followed by Bonferroni post-test and represented as mean ± s.e.m (*P < 0.05; **P < 0.01; ***P < 0.001).

So far, over 150 mutations in the primary sequence of TTR have been identified, most of which are associated with amyloidosis (4, 28). Therefore, we questioned if other mutated TTR variants besides V30M also failed to activate SCs. For that, SCs were incubated with WT TTR as a control, I68L TTR (a cardiomyopathy mutation), T119M TTR (a non-pathological mutation) and I84S (a mutation that leads to carpal tunnel syndrome and affects heart and eye) for 24 hours and assessed the expression levels of several chemokines by real-time PCR, as before. The I84S mutation also serves as a control since it has lower affinity for the TTR-thyroxine and TTR-RBP complexes (29) serving to rule out the effect of these ligands in the assays. TTR molecules carrying other pathological mutations (I68L and I84S) or the non-pathological mutation T119M activated SCs, leading to an expression of chemokines globally similar to that induced by WT TTR (**Figure 3**). The expression of IL-6 was also similar between SCs incubated with WT or mutant TTRs (**Figure 3**). Altogether, these results indicate that in contrast with V30M TTR, other mutations in the TTR protein did not impair SCs activation to induce chemokine expression.

### TLR4 Mediates the Expression of Chemokines in Schwann Cells Incubated With WT TTR

**Figure 4:**
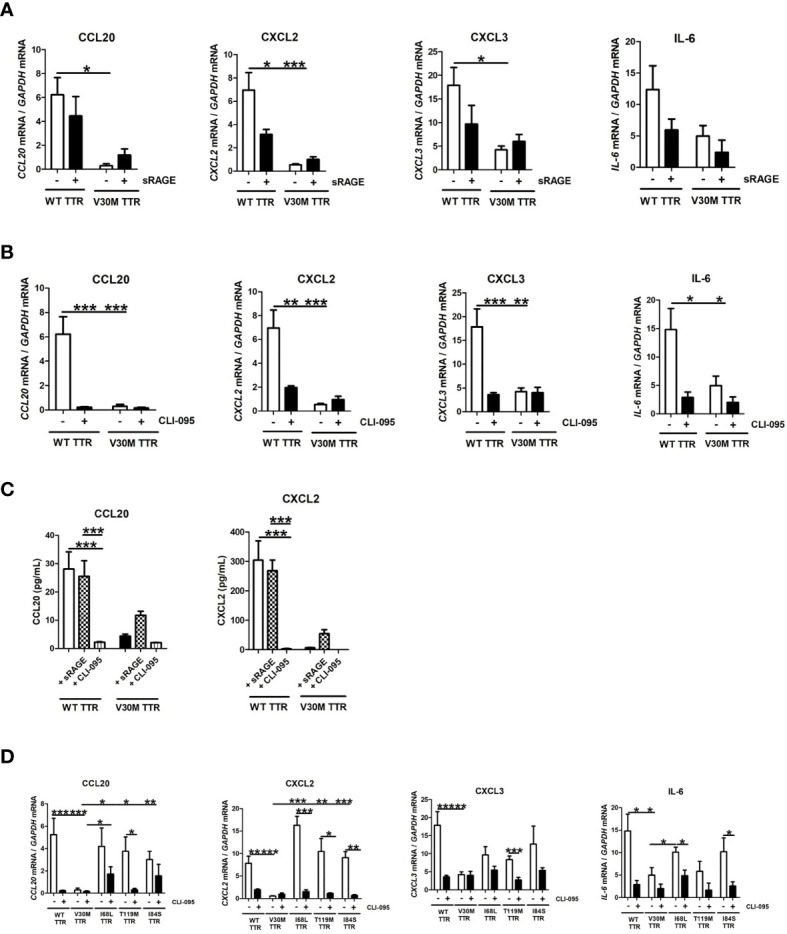
WT TTR activates TLR4 signaling pathway and V30M TTR fail to do so. RNA was extracted from SCs incubated with WT and V30M TTR in the presence of TLR4 **(A)** and RAGE **(B)** antagonist and denoted a downregulation in the expression of several chemokines in SCs incubated with WT TTR but not in SCs incubated with V30M TTR. **(C)** Decreased concentration levels of CCL20 and CXCL2 in the supernatant of SCs incubated with WT TTR in the presence of TLR4 antagonist. **(D)** SCs were incubated with pathological and non-pathological mutations in the presence of TLR4 antagonist and the inhibition of this receptor reduces the expression of some chemokines. Data were analyzed using one-way ANOVA followed by Bonferroni post-test and represented as mean ± s.e.m (*P < 0.05; **P < 0.01; ***P < 0.001).

Several studies suggest a role for RAGE receptor in mediating TTR signaling mechanisms (12, 30). Thus, we investigated the role of RAGE in mediating the activation of SCs by TTR. Inhibition of the RAGE receptor with an antagonist (sRAGE) did not significantly impact the expression of chemokines or of IL-6 by SCs incubated with WT TTR (**Figure 4A**). Since RAGE interacts with TLR4 (31, 32), we next questioned whether TLR4 may itself play a role in the induction of chemokines by SCs incubated with TTR. TLR4 inhibition with the antagonist CLI-095 highly reduced the expression of chemokines and of IL-6 induced by WT TTR stimulation of SCs (**Figure 4B**). As before, V30M TTR only induced residual expression of the tested genes, which was not altered by the presence of the TLR4 inhibitor (**Figure 4B**). The expression data for CCL20 and CXCL2 were further validated at the protein level (**Figure 4C**). Collectively, our findings indicate that WT TTR triggers TLR4 signaling in SCs thus leading to the expression and secretion of several chemokines.

Given these findings, we then questioned if TLR4 also mediated the activation of SCs by other TTR mutations. For that, we incubated SCs with WT, I68L, T119M or I84S in the presence of antagonists for TLR4 and RAGE as above. Overall, the TLR4 inhibition by CLI-095 reduced the expression of all tested chemokines in SCs incubated with all mutant forms of TTR tested (**Figure 4D**). We also measured the cytokine IL-6 with similar results (**Figure 4D**).

## Discussion

In the present study, we showed an impairment in the production of several chemokines in the peripheral nervous system of a pre-clinical model of TTR V30M amyloidosis. Additionally, we demonstrate that WT TTR activates the TLR4 receptor in cultured SCs contrariwise to V30M TTR. We consequently hypothesize that SCs are compromised in their function in ATTRv patients, which may explain the downregulated expression of several chemokines and the lack of inflammatory infiltrates observed in peripheral nerves.

The inflammatory response has been shown to be important for several neurodegenerative disorders (33). Immune responses are typically mediated by immune cells such as antigen-presenting cells, macrophages or T-cells. However, also non-immune cells such as SCs in the PNS might play a key role in innate and adaptive immune responses (14, 34). Regarding to ATTRv, despite cytokine production by axons, no influx of inflammatory cells is found in patients nerve biopsies, suggesting that mechanisms must operate to prevent the correct innate immune response (13, 35). Consistent with this observation, our laboratory previously showed that in V30M mouse model, 7 days post sciatic nerve injury, neutrophil and macrophage infiltration is lower when compared to WT mice, which is indicative of a specific phenotype associated with the V30M mutation. We hypothesized that a non-optimal activation of SCs occurred since, after injury, decreased expression of pro-inflammatory cytokines and chemokines was observed, thus culminating in a diminished immune cellular activation and infiltration in V30M nerves (36). Likewise, in nerves of ATTRv patients, SCs are impaired in their ability to express chemokines and neurotrophic factors important to drive tissue regeneration, contributing this way for neuronal dysfunction present in the disease (12). In line with previous findings, our data demonstrate that several chemokines such as CCL20, CCL8, CCL5, CXCL5, CCL2, CXCL2 and CXCL3 were highly downregulated in HSF V30M TTR mice in both peripheral nerves and plasma samples, pointing towards a close interface between the absence of chemokine response and cellular infiltration with the development of the disease. Chemokines are also produced by other cells than SC. The fact that lower levels of chemokines were observed in circulation may hint at differences in the response of other cell types (in addition to SC) to WT or V30M TTR. Future studies addressing this hypothesis are needed.

It will be important to address the differential chemokine production in human patients. Our preliminary data obtained for different chemokines in the human plasma suggested only minor differences between a small group of ATTRv patients and controls (data not shown). However, this apparent contradiction with the animal data may be explained by several reasons. Firstly, further studies on a greater number of ATTRv blood samples from carriers of different TTR mutations are required to strengthen the results obtained so far and, ultimately, to evaluate whether blood levels of CCL20 or another chemokine can be used as a possible biomarker for ATTRv. Secondly, by measuring the total levels of circulating chemokines, we may be overcoming a possible local downregulation observed in the sciatic nerve. Additionally, in contrast to transgenic mice and cultured SCs, the vast majority of ATTRv patients are heterozygous for the mutation and, therefore lower levels of WT TTR are present in circulation. Even lower than normal levels of WT TTR may be sufficient to stimulate the TLR4 receptor correctly. Therefore, in future studies, it will be interesting to understand if the addition of WT TTR can rescue the V30M TTR phenotype. Recent studies suggests that ATTRv patients have altered levels of several cytokines in the serum, some of them involved in pro- and anti-inflammatory response (36, 37). In fact besides neuropathic condition, patients with ATTRv also present gastrointestinal symptoms, cachexia, malnutrition, diarrhea and others symptoms (38).

SCs play an important role in immune surveillance and have the capacity to detect pathogens and to orchestrate an ensuing immune response (39). Charcot–Marie–Tooth disease, Guillain–Barré syndrome, schwannomatosis and chronic inflammatory demyelinating polyneuropathy are all neuropathies involving SCs (40). It is well known that SCs differentiation and migration plays a critical role to guide and support axonal growth (41, 42) and, in ATTRv patients, SCs are impaired in their ability to express chemokines and neurotrophic factors important to drive tissue regeneration, contributing in this way for neuronal dysfunction present in the disease (12). Taken together the fact that TTR gene is expressed in SCs suggesting a specific role of TTR in the peripheral nervous system, such as SC–neuron interaction (7, 43) and the compromised nerve regeneration after injury in the transgenic V30M mouse model (17), we decided to investigate the role of SCs in ATTRv immune response. Interestingly, we found that SCs are activated by incubation with WT TTR contrariwise to incubation with V30M TTR and respond with the production of several chemokines and cytokines. Additionally, we also observed an increase in the phosphorylation of MAP kinase p38 in SCs incubated with WT TTR. p38 mediates inflammatory responses partly through activating gene expression. Proteins phosphorylated by a mechanism dependent on MAP kinase p38 activity include sequence-specific transcription factors, transcriptional regulators, nucleosomal proteins, and regulators of mRNA translation (44). The lower phosphorylation of p38 in SCs incubated with V30M TTR could help to explain the lack of inflammatory cells observed in ATTRv patient’s nerve biopsies. However, additional studies are required to further investigate this finding.

Moreover, we implicate TLR4 as the receptor being triggered by TTR in SCs. It was previously shown that neurite outgrowth was impaired in DRG neurons from TTR-knockout mice, and the addition of WT TTR could rescue that condition (45). Thus, WT TTR secreted from SCs might play important roles in maintaining the microenvironment of the peripheral nervous system necessary for nerve regeneration (45, 46). Contrariwise, SCs incubated with V30M TTR do not respond with the expression of several chemokines important for the chemoattraction of macrophages, neutrophils and other immune cells. This may be the reason underlying the lack of significant inflammatory infiltrates in the nerves of animal models and ATTRv patients.

The reason why only V30M TTR variant fails to activate SCs remains unclear and additional studies are required to unveil and understand the molecular pathways underlying this observation. However, it is tempted to speculate that only the neuropathic form of TTR fails to activate SCs. To further understand the activation of SCs by TTR variants, we incubate these cells with other pathological and non-pathological mutated forms of TTR. Interestingly, all tested variants triggered the expression of chemokines in SCs *via* TLR4 globally similar to what was observed for WT TTR. It is possible that the V30M mutation might acquire a molecular or conformational specificity that impairs the activation of SCs, leading to disease progression. Independently of the mechanism, the dissection of the cellular pathways involved in this disease is of greatest importance for the enhancement of new treatment approaches, which as our findings suggest may need to be adjusted to the mutation present in TTR.

Taking into consideration the role of WT TTR in the activation of SCs and consequential expression of chemokines, it would be interesting in the future to investigate if TTR may also trigger a chemokine response in other innate immune cells, such as macrophages, the main phagocytic cells of the immune system. In fact, ATTRv patients display quantitative and qualitative abnormalities in macrophages with defects in cell adhesion and chemotaxis (47). Additionally, it was recently demonstrated that the number of heart resident macrophages is significantly decreased in ATTRv patients as compared with healthy donors (48). Therefore, the possible role of TTR in inflammation and immune response, as well as the underlying mechanisms, needs to be further elucidated.

Overall, we show that WT TTR activates SCs *via* TLR4 receptor leading to the expression of several chemokines. Critically, V30M TTR does not activate SCs thus failing to induce the expression of chemokines. How these mediators interact in the context of ATTRv may be crucial to explain the immunological impairment observed in peripheral nerves of ATTRv patients, because all of these observations suggest that V30M TTR mutation may be responsible for modify the function of certain signaling pathways.

## Data Availability Statement

The datasets presented in this study can be found in online repositories. The names of the repository/repositories and accession number(s) can be found below: NCBI Gene Expression Omnibus, accession no: GSE165126.

## Ethics Statement

The animal study was reviewed and approved by Sofia Lamas, i3S.

## Author Contributions

JM planned, executed and wrote the manuscript. SC executed some experiments and approved final manuscript. MS planned, reviewed experiments, and corrected manuscript. MJS planned and review experiments and corrected manuscript. All authors contributed to the article and approved the submitted version.

## Funding

The work was funded by the project Norte-01-0145-FEDER-000008 - Porto Neurosciences and Neurologic Disease Research Initiative at I3S, supported by Norte Portugal Regional Operational Programme (NORTE 2020), under the PORTUGAL 2020 Partnership Agreement, through the European Regional Development Fund (FEDER). JM was supported by FCT with a PhD fellowship SFRH/BD/129345/2017. MS is funded by FCT through Estímulo Individual ao Emprego Científico.

## Conflict of Interest

The authors declare that the research was conducted in the absence of any commercial or financial relationships that could be construed as a potential conflict of interest.

## Abbreviations

FAP, Familial amyloidotic polyneuropathy; Gapdh, Glyceraldehyde 3-phosphate dehydrogenase; Hsf-1, Heat shock factor 1; PBS, Phosphate buffer saline; PNS, Peripheral nervous system; RT-PCR, Real-time PCR; SEM, Standard error of the mean; TTR, Transthyretin; WT, Wild-type; ELISA, enzyme-linked immunosorbent assay; BMDM, bone marrow derived macrophages; SDS, sodium dodecyl sulphate; PAGE, polyacrylamide gel electrophoresis.
